# Hydrolysis products of agricultural waste can serve as microbial fertilizer enhancers to promote the growth of maize crops

**DOI:** 10.3389/fpls.2024.1405527

**Published:** 2024-10-17

**Authors:** Yu Xu, Wei Wang, He Wang, Yinping Tian, Zhengfu Yue, Cheng Li, Yuefeng Wang, Jing Zhang, Ruifu Zhang

**Affiliations:** ^1^ Key Laboratory of Agricultural Water Resources, Hebei Key Laboratory of Soil Ecology, Center for Agricultural Resources Research, Institute of Genetics and Developmental Biology, Chinese Academy of Sciences, Shijiazhuang, China; ^2^ Jiangsu Provincial Key Lab for Organic Solid Waste Utilization, National Engineering Research Center for Organic-based Fertilizers, Jiangsu Collaborative Innovation Center for Solid Organic Waste Resource Utilization, Nanjing Agricultural University, Nanjing, China; ^3^ Key Laboratory of Low-carbon Green Agriculture in Tropical region of China, Ministry of Agriculture and Rural Affairs; Hainan Key Laboratory of Tropical Eco-Circular Agriculture, Environment and Plant Protection Institute, Chinese Academy of Tropical Agricultural Sciences, Haikou, China; ^4^ Hebei Provincial Laboratory of Water Environmental Science, Hebei Provincial Academy of Ecological and Environmental Sciences, Shijiazhuang, China; ^5^ Department of Environmental Sciences, School of Tropical and Laboratory Medicine, Hainan Medical University, Haikou, China

**Keywords:** agricultural wastes, organic acids, hypoxia hydrolysate, PGPR, root

## Abstract

Efficient utilization of agricultural wastes and reduction of chemical fertilizer inputs are crucial for sustainable development of agriculture. Plant growth promoting rhizobacteria (PGPR) are widely used as biofertilizers to partially replace chemical fertilizers in agricultural production. The functional performance of PGPR strains is closely related to their root colonization capacity. Some organic acids from root exudates can recruit PGPR to colonize the root. In this study, agricultural organic wastes such as mushroom bran and tobacco waste materials were used to produce organic acids through the hypoxic hydrolysis process. The hydrolysis conditions were optimized to maximize the production of a mixture of complex organic acids from the hypoxic hydrolysis of these materials, employing both single-factor and orthogonal experimental methods. The diluted hydrolysates were tested for their effects on the rhizosphere colonization of the PGPR strain *Bacillus amyloliquefaciens* SQR9 using fluorogenic quantitative PCR in greenhouse pot experiments. The results demonstrated that hypoxic hydrolysates from tobacco waste and mushroom bran significantly enhanced the colonization of SQR9 in the maize rhizosphere. Specifically, a 2000-fold dilution of tobacco waste hydrolysate yielded the most effective result, while a 5000-fold dilution of mushroom bran hydrolysate provided the best outcome. All treatments combining these hydrolysates with SQR9 significantly increased maize stem dry weight, indicating that with appropriate treatment, such as anaerobic fermentation, these agricultural organic wastes can serve as synergistic agents of microbial fertilizers, contributing to agricultural sustainability.

## 1 Introduction

Chemical fertilizers and pesticides have greatly contributed to the development of agriculture, but their overuse has also led to serious environmental damage. This includes the degradation of soil and the quality of agricultural products, which has restricted the sustainable development of agriculture (Ding et al., 2024; Wang et al., 2022, 2024; Wu et al., 2023). Another issue in modern agriculture is the management and efficient utilization of the large amount of agricultural waste, such as crop straws and by-products from agricultural product processing. Discarding these organic materials causes environmental pollution and a waste of valuable resources. However, if properly treated, these materials can be used to improve soil quality and agricultural production (Koul et al., 2022).

Plant growth-promoting rhizobacteria (PGPR) play important roles in sustainable agricultural production (Kunal et al., 2023). They promote plant growth by producing phytohormones and releasing essential nutrients such as phosphorus and potassium (Shao et al., 2015). PGPR can also suppress soil-borne diseases by occupying rhizosphere niches and exhibiting antagonistic properties (Li et al., 2014; Liu et al., 2014; Zhang et al., 2015). With growing concern over the negative effects of chemical fertilizers and pesticides on the environment and soil, PGPR is becoming increasingly popular in agricultural production due to its low cost, high efficiency and environmentally friendly nature. It has the potential to reduce the use of chemical fertilizers and pesticides.

It is well known that efficient root colonization of PGPR is necessary for them to exert their plant beneficial effects, and root colonization is related to the chemotaxis ability of PGPR towards root exudates (Guo et al., 2024). Many studies have reported that low molecular weight organic acids in root exudates play a role in recruiting PGPR for root colonization. For example, L-malic acid (MA) secreted from roots recruits the beneficial rhizobacterium *Bacillus subtilis* FB17 (Yang et al., 2023). Additionally, root-secreted citric acid and fumaric acid positively influence the colonization of *Bacillus amyloliquefaciens* SQR9 in the cucumber rhizosphere (Liu et al., 2014). Furthermore, several root-secreted organic acids can recruit *Paenibacillus polymyxa* SQR-21 (Ling et al., 2011). Therefore, to increase root colonization and the beneficial efficiency of PGPR, it is possible to produce low molecular weight organic acids as auxiliary agents or biofertilizers.

The hydrolysis reaction is the early stage of the anaerobic fermentation process, which can hydrolyze macromolecular organic substances such as agricultural organic waste into low molecular weight organic acids without strict anaerobic conditions (Stams et al., 2006; Wang et al., 2009; Weiland, 2010). Mushroom bran and tobacco waste contain rich organic substances, including carbohydrates, proteins, and cellulose. These components can be converted into low molecular weight organic acids during the hydrolysis process (Ketemepi et al., 2024; He et al., 2024). Therefore, producing organic acids through the hydrolysis of agricultural organic waste, such as mushroom bran and tobacco waste, and then using these hydrolysis products to enhance the root colonization of PGPR, not only provides a new way for the utilization of agricultural waste, but also offers a new strategy to boost the efficacy of PGPR.

Maize is the largest cultivated crop in China and a vital food source for its population (Yue et al., 2022). However, the current maize yield in China is only 60% of that in developed countries (Guo et al., 2021). A key factor contributing to this gap is the degradation of soil structure and nutrient imbalances resulting from the prolonged overuse of chemical fertilizers and pesticides. To fully leverage the growth-promoting effects of PGPR on maize yield, this study optimized the hypoxic hydrolysis process of mushroom bran and tobacco waste. The hydrolysates obtained from these processes were then applied in conjunction with *Bacillus amyloliquefaciens* SQR9, to enhance root colonization and promote growth in maize. The integration of these hydrolysates with SQR9 aimed to significantly improve maize yield by fostering more robust plant-microbe interactions.

## 2 Materials and methods

### 2.1 Strains and substrates

*Bacillus amyloliquefaciens* SQR9 (CGMCC accession no. 5808, China General Microbiology Culture Collection Center) is a well-studied and widely used PGPR strain (Cao et al., 2011; Qiu et al., 2014; Xu et al., 2013). It was cultured in LB medium (10 g/L tryptone, 5 g/L yeast extract, 3 g/L NaCl) (Shao et al., 2015). The mushroom bran and tobacco waste utilized for hypoxic hydrolysis were sourced from the Forestry Bureau in Tonghe County, Harbin, Heilongjiang Province (45.98, 128.75), and the Hongyun Honghe Group’s Qujing Cigarette Factory (25.53, 103.81), respectively. The mushroom bran had a moisture content of 9.3%, a pH of 6.5, and a C/N ratio of 42.6, while the tobacco waste had a moisture content of 7.2%, a pH of 7.2, and a C/N ratio of 35.9. The inoculated sludge used to initiate the fermentation of hydrolysis was obtained from the sediments of the local Qinhuai River (32.01, 118.83).

### 2.2 Optimization of the hypoxic hydrolysis

Single factor experiments for the optimization of organic acid production conditions were firstly conducted. The hypoxic hydrolysis was carried out in a brown bottle (500 mL) with its mouth sealed by a leather stopper. Mushroom bran or tobacco waste materials were air-dried, passed through a 2 mm sieve and autoclaved. Then, they were added into the bottle (34.5 g in 500 mL). Five factors were designed for the optimization, including initial pH, temperature, inoculum, moisture content and hydrolysis time, respectively. Each factor had four levels: 5.0, 6.0, 7.0, and 8.0 for pH; 30°C, 35°C, 40°C, and 45°C for temperature; 5%, 10%, 15%, and 20% (w/w) for inoculum; and 80%, 85%, 90%, and 95% for moisture content. The hydrolysis lasted for 7 days and samples were taken every 12 hours.

In the orthogonal experiment, two optimal levels were included for each of the above single factors, resulting in a total of 5 factors and 2 levels (**Supplementary Tables S1**, **S2**).

### 2.3 Measurement of organic acids

Organic acids were determined by the colorimetric method (Montgomery et al., 1962). Briefly, the hydrolysis supernatant was obtained by centrifuging the sample at 10000 rpm, and then 0.5 mL of the supernatant was transferred into a tube. 1.7 mL of reagent B (30 mL of ethylene glycol with 4 mL of dilute sulfuric acid and an equal volume of water) was added to the tube and mixed thoroughly. The tube was heated in a boiling-water bath for 3 min, and then cooled down in an ice water bath immediately. Subsequently, 2.5 mL of reagent C (20 mL of 4.5 mol/L sodium hydroxide with 5 mL of hydroxylamine sulphate) was added, mixed and settled aside for 1 minute. The solution was then transferred into a 25 mL calibrated flask, and 10 mL of reagent D (dissolve 20 g of ferric chloride hexahydrate in 500 mL of water, add 20 mL of concentrated sulfuric acid, and dilute to 1 liter) was added. The optical density at a wavelength of 500 nm was measured with distilled water as the blank control. The concentrations of organic acids produced by the hypoxic hydrolysis process were calculated using the following equation:


(1) $$C=C_{0}\times V_{d}/V$$

Where *C* (mg/mL) is the concentration of total organic acids, *C_0_* (mg/mL) is the optical density at 500 nm wavelength corresponding to the amount of organic acid in the acetic acid standard curve, *V_d_* is the dilution factor of the measurement, and *V* is the volume of the samples (mL).

### 2.4 Pot experiments

The pot experiments were conducted in the greenhouse on the campus of Nanjing Agricultural University, located in Nanjing, Jiangsu Province, China (118.78°E, 32.05°N). During the experiment, the temperature in the greenhouse was maintained between 20°C and 28°C, with a light transmittance of 50-60% and humidity levels ranging from 55-85%. Water supply was manually controlled to ensure consistent and adequate irrigation, with no influence from natural rainfall. Surface soil (0-20 cm) was collected from the Baima Teaching and Research Base of Nanjing Agricultural University (119.17°E, 31.63°N). The area has a subtropical monsoon climate, featuring hot, humid summers and mild winters. The average annual temperature ranges from 15°C to 16°C, with most of the 1000-1200 mm of annual precipitation occurring during the summer months. The soil type was yellow-brown soil, iron wet leaching soil, with properties including a pH of 8.18, organic matter of 5.46 g/kg, total nitrogen of 1.27 g/kg, available phosphorus of 6.8 mg/kg, and available potassium of 88.4 mg/kg. The soil was air-dried, ground, and passed through a 2 mm sieve, then mixed with quartz sand (particle size 0.106-0.212 mm) in a 7:3 ratio. This mixture helps prevent soil compaction and improves the soil’s water and air permeability. Each pot contained 1.5 kg of soil mixture. Maize (“Jingtian” purple waxy maize) seeds were surface-sterilized with 0.1% NaClO and 70% ethanol, germinated and grown for one week. Then, the maize seedlings were transplanted into the pot, with one seedling in each pot. All hypoxia hydrolysates were sterilized using a sterile membrane of 0.22 μm in diameter before being added to the pot. Eleven treatments were included in the pot experiment with the addition of different agents as follows: 1) 50 mL of sterile distilled water served as the blank control (Blank); 2) 50 mL of 1000-times diluted mushroom bran hypoxia hydrolysate (J1000); 3) 35 mL of 1000-times diluted mushroom bran hypoxia hydrolysate plus inactivated SQR9 (J1000+SQR9(I)); 4) 35 mL of 1000-times diluted mushroom bran hypoxia hydrolysate plus living SQR9 (J1000+SQR9); 5) 35 mL of 2000-times diluted mushroom bran hypoxia hydrolysate plus living SQR9 (J2000+SQR9); 6) 35 mL of 5000-times diluted mushroom bran hypoxia hydrolysate plus living SQR9 (J5000+SQR9); 7) 50 mL of 1000-times diluted hypoxia hydrolysate of tobacco waste materials (Y1000); 8) 35 mL of 1000-times diluted hypoxia hydrolysate of tobacco waste materials plus inactivated SQR9 (Y1000+SQR9(I)); 9) 35 mL of 1000-times diluted hypoxia hydrolysate of tobacco waste materials plus living SQR9 (Y1000+SQR9); 10) 35 mL of 2000-times diluted hypoxia hydrolysate of tobacco waste materials plus living SQR9 (Y2000+SQR9); 11) 35 mL of 5000-times diluted hypoxia hydrolysate of tobacco waste materials plus living SQR9 (Y5000+SQR9). The dilution ratio of the hydrolysate was determined based on the results of the chemotaxis assay (**Supplementary Figure S1**). SQR9 was cultured for 24 hours at 35°C, and the concentration of SQR9 in the culture was 5×10^8^ CFUs/mL. SQR9 cells were obtained by centrifugation, and resuspended in the same volume of water. 15 mL of the SQR9 suspension was added to each pot. Inactivated SQR9 was obtained by autoclaving at 121°C for 2 hours. Ten replicates were included for each treatment, which were randomly blocked. Maize and rhizosphere soil were sampled after 7 and 14 days, respectively. The pot experiment was concluded after the collection on the 14th day.

### 2.5 Analysis of SQR9 abundance in maize rhizosphere

#### 2.5.1 Collection of rhizosphere soil

The excess soil on the roots was gently shaken off, and then these roots were transferred into a sterile 50 mL centrifuge tube containing PBS-S buffer (6.33 g/L NaH_2_PO_4_·H_2_O, 16.5 g/L Na_2_HPO_4_·7 H_2_O, 200 μL/L Silwet L-77). The tubes were shaken at 180 rpm for 20 min and then vortexed at maximum speed for 15 seconds before being centrifuged at 5500 g for 5 min to remove the supernatant. The remaining rhizosphere soil was used to extract DNA.

#### 2.5.2 Quantification of SQR9 by RT-qPCR

The rhizosphere soil DNA was extracted using the FastDNA^®^ SPIN Kit for Soil (Mpbio, America), strictly following the steps in the instructions. Specific primers for SQR9 were designed targeting a unique genomic island of SQR9 (Wang et al., 2019), which were F 5’-ATAGCAAGAGCGAGGCAGAAGT-3’ and R 5’-CAGAGGAATCATCAACACCAACAGT-3’. RT-qPCR amplification was performed on the Applied Biosystems 7500 Real-Time PCR system using the Premix Ex Taq™ kit (Takara, Dalian, China). The PCR reaction mixture contained 10.0 μL of Premix Ex TaqTM (2×), 0.4 μL of each primer (10 μmol/L), 0.4 μL of ROX Reference Dye II (50×), 20 ng of DNA template, and a final volume of 20 μL with sterile water. The PCR program consisted of an initial denaturation at 95°C for 30 s, followed by 40 cycles of denaturation at 95°C for 5 s and annealing and extension at 60°C for 34 s. The threshold cycle (Ct) values were automatically calculated by the system. The melt curve analysis was conducted at the end of the PCR run to evaluate amplification specificity. Before that, a standard curve was created according to the above procedure.

### 2.6 Maize growth analysis

Plant height and shoot dry weight were measured for three randomly selected maize seedlings from each treatment. Due to the short growth cycle set for the maize plants, a significant portion of the root system was lost during the collection of sufficient rhizosphere soil; therefore, root biomass data were not recorded.

### 2.7 Statistical analysis

Differences among the treatments were calculated and statistically analyzed with a one-way analysis of variance (ANOVA). Duncan’s multiple range test was used when the one-way ANOVA indicated significant differences (*p<* 0.05). All statistical analyses were carried out with SPSS BASE ver.11.5 statistical software (SPSS, Chicago, IL, USA).

## 3 Results

### 3.1 Hydrolysis of the agricultural wastes for organic acids production

To produce organic acids through hydrolysis from agricultural wastes such as mushroom bran and tobacco waste materials, the factors affecting hydrolysis efficiency were investigated to obtain the optimal reaction parameters. These factors include the initial pH, temperature, sediment inoculum, moisture and reaction time.

**Figure 1:**
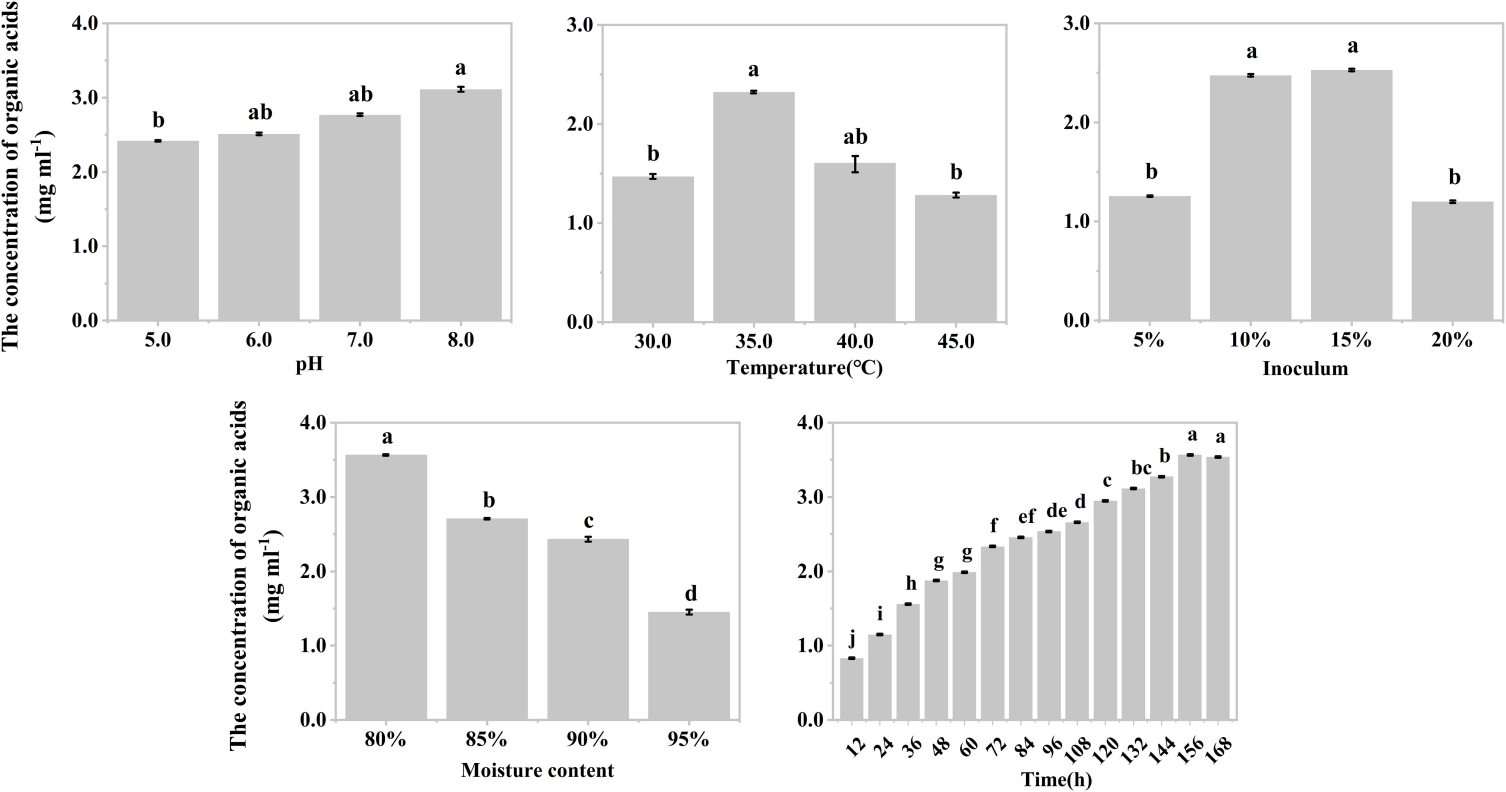
Organic acids production in different reaction conditions with mushroom bran in single factor experiment. Significant differences among the different treatments are indicated by different letters (*p<* 0.05).

**Figure 2:**
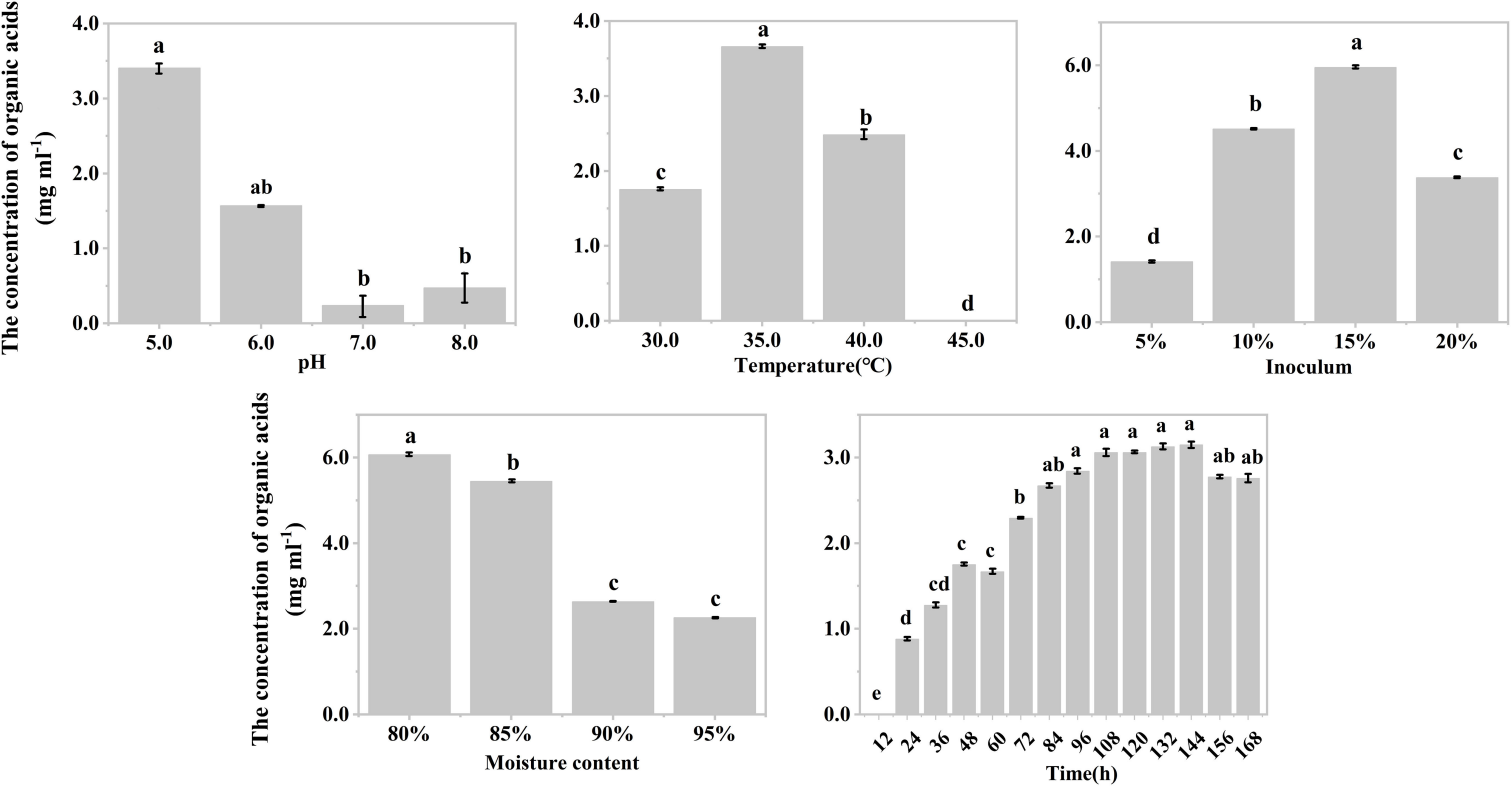
Organic acids production in different conditions with tobacco wastes in single factor experiment. Significant differences among the different treatments are indicated by different letters (*p<* 0.05).

The effects of temperature, inoculum, and moisture on the production of organic acids during the hypoxic hydrolysis of mushroom bran and tobacco waste were largely consistent. Specifically, the concentration of produced organic acids initially increased and then decreased with rising incubation temperature (ANOVA, mushroom bran: *p*< 0.001; tobacco waste: *p*< 0.001) and inoculum (ANOVA, mushroom bran: *p*< 0.001; tobacco waste: *p*< 0.001), while it gradually decreased with increasing moisture (ANOVA, mushroom bran: *p*< 0.001; tobacco waste: *p*< 0.001) (**Figures 1B–D**, **2B–D**; **Supplementary Table S3**). However, the effects of initial pH and incubation time on organic acid production differed between the two substrates. For mushroom bran, the organic acids gradually increased with a higher initial pH (ANOVA, *p*< 0.001), whereas for tobacco waste, it exhibited the opposite trend (ANOVA, *p*< 0.001) (**Figures 1A**, **2A**). Additionally, organic acid production from mushroom bran increased with incubation time (ANOVA, *p*< 0.001), reaching a plateau at 156 h, while for tobacco waste, it initially increased and then decreased with prolonged incubation time (ANOVA, *p*< 0.001) (**Figures 1E**, **2E**).

In summary, for the hydrolysis of mushroom bran, the optimal initial pH, temperature, inoculum, moisture and time were 8.0, 35°C, 10%, 80% and 156 h, respectively. For the hydrolysis of tobacco waste, the optimal initial pH, temperature, inoculum, moisture and time were 5.0, 35°C, 15%, 80% and 144 h, respectively.

**Figure 3:**
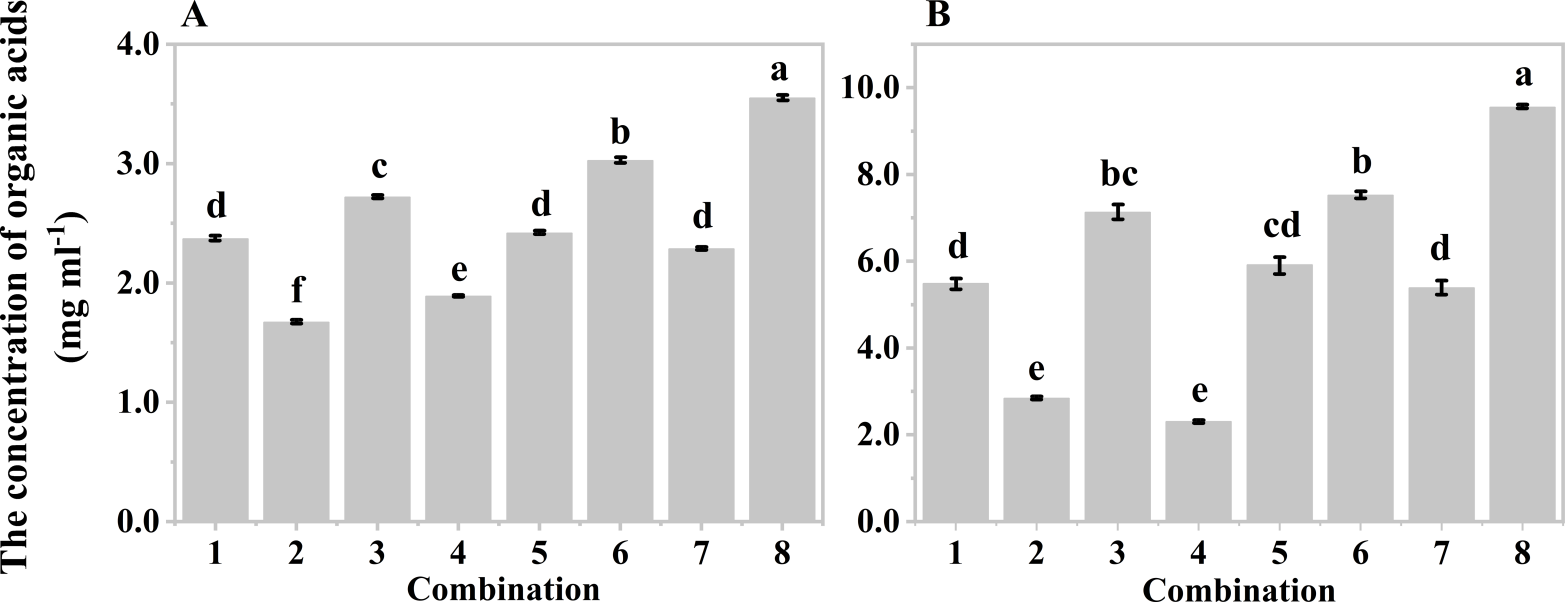
Organic acids production of different combinations in orthogonal experiment: **(A)** mushroom bran hypoxia hydrolysis; **(B)** tobacco wastes hypoxia hydrolysis. Significant differences among the different treatments are indicated by different letters (*p<* 0.05).

Orthogonal experiments were also conducted to determine the optimal hydrolysis conditions for producing organic acids from both wastes. Five-factor two-level orthogonal experiments were designed based on the results of the single factor experiments, resulting in eight different combinations for mushroom bran (**Supplementary Table S1**) and tobacco waste materials (**Supplementary Table S2**). Since the system with a moisture content of 80% was too viscous for solid-liquid separation after hydrolysis, moisture contents of 85% and 90% were used in the orthogonal design. Both mushroom bran (**Figure 3A**) and tobacco waste materials (**Figure 3B**) showed the highest production of organic acids in combination No. 8 of **Supplementary Tables S1**, **S2**. Therefore, the optimal hypoxia hydrolysis conditions were an initial pH of 8.0, a temperature of 40°C, a sediment inoculum of 15%, a moisture content of 85%, and a hydrolysis time of 168 h for mushroom bran. For tobacco waste materials, the optimal conditions were an initial pH of 6.0, a temperature of 40°C, a sediment inoculum of 15%, a moisture content of 85%, and a hydrolysis time of 144 h.

### 3.2 Effect of hydrolysate on maize rhizosphere colonization of PGPR strain SQR9

**Figure 4:**
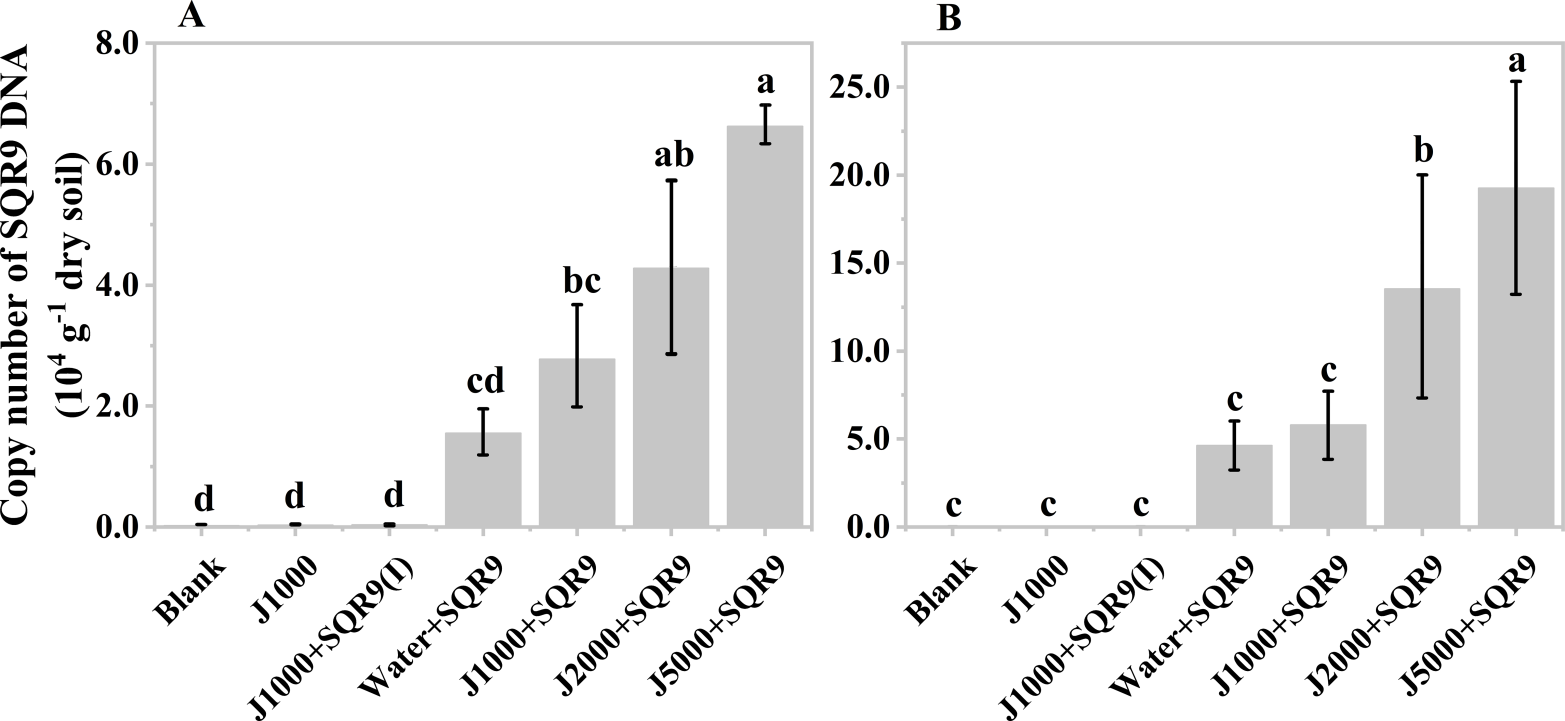
Copy number of SQR9 in different treatments with mushroom bran hypoxia hydrolysate dilutions after 7 days **(A)** and 14 days **(B)** of planting. Significant differences among the different treatments are indicated by different letters (*p<* 0.05).

After obtaining the organic acids from the hydrolysis of mushroom bran and tobacco waste materials, their effect on the rhizosphere colonization of the PGPR strain SQR9 was evaluated. The results showed that diluted mushroom bran hydrolysates increased the maize rhizosphere colonization of SQR9 at both sampling times (7 and 14 days after planting) (**Figures 4A, B**). Compared to the water control, the diluted mushroom bran hydrolysate significantly (ANOVA, *p*< 0.001) enhanced SQR9 colonization in the maize rhizosphere, with the effect increasing alongside higher dilution factors (**Figures 4A, B**; **Supplementary Table S3**). This result is consistent with the findings from the chemotaxis assay conducted *in vitro* (**Supplementary Figure S1**), demonstrating that the enhancement of SQR9 colonization by the organic acids in the mushroom bran hydrolysate depends on an appropriate concentration range. The 5000-fold dilution of the mushroom bran hydrolysate provided the optimal enhancement of SQR9 colonization in the maize rhizosphere (**Figures 4A, B**).

As expected, the blank, 1000-times diluted hydrolysate alone, and 1000-times diluted hydrolysate with inactivated SQR9 controls did not detect SQR9 colonization, demonstrating the specificity of the q-PCR primers for SQR9 (Qiu et al., 2014). Additionally, compared to 7 days after maize planting (**Figure 4A**), the SQR9 colonization in the maize rhizosphere was 2-4 times higher after 14 days (**Figure 4B**). This indicates that SQR9 exhibits ongoing chemotactic activity towards the rhizosphere throughout maize growth.

**Figure 5:**
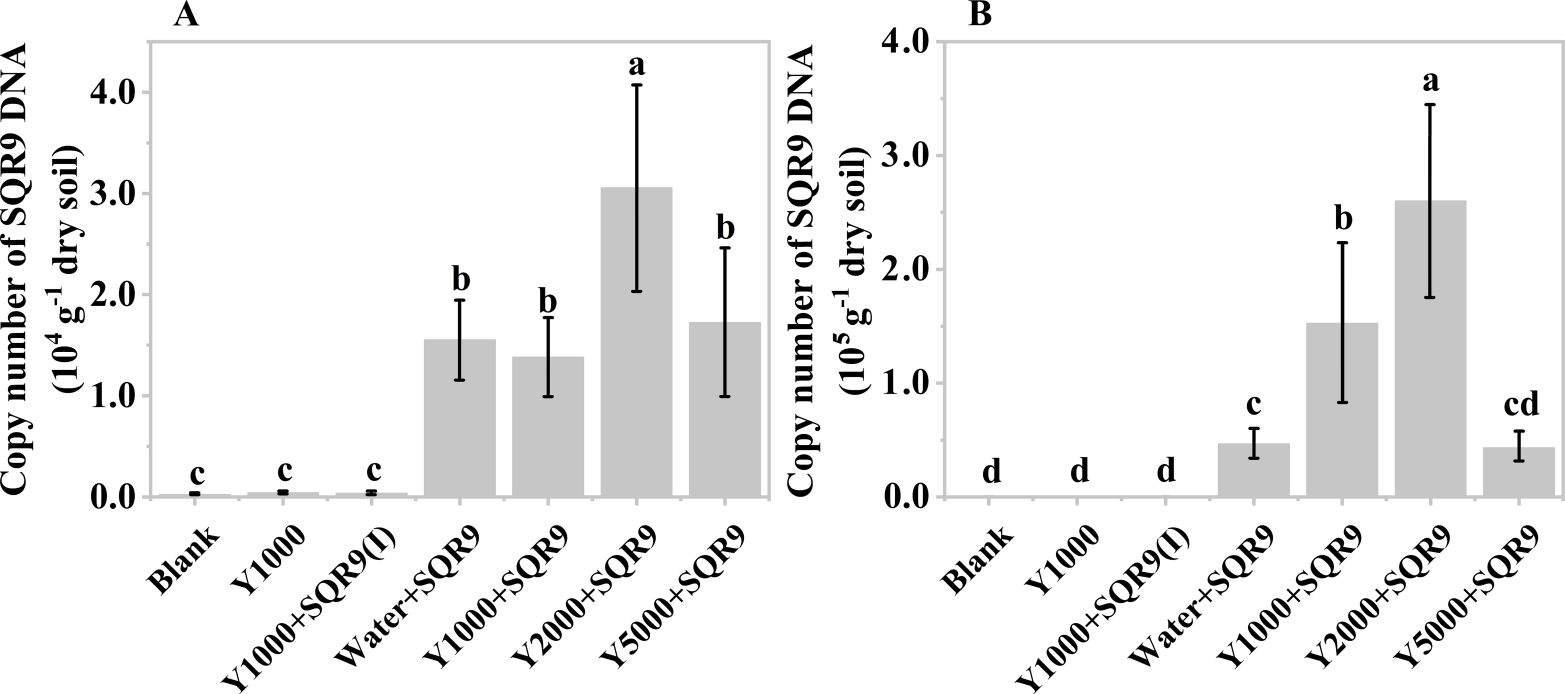
Copy number of SQR9 in different treatments with hypoxia hydrolysate dilutions of tobacco wastes after 7 days **(A)** and 14 days **(B)** of planting. Significant differences among the different treatments are indicated by different letters (*p<* 0.05).

The diluted hydrolysate of tobacco waste materials showed a similar effect on SQR9 rhizosphere colonization (**Figures 5A, B**). However, unlike the mushroom bran hydrolysate (**Figures 4A, B**), the 2000-fold diluted tobacco waste hydrolysate exhibited the highest enhancement of SQR9 colonization at both maize sampling times (**Figures 5A, B**), which was consistent with the chemotaxis experiment results *in vitro* (**Supplementary Figure S1**). These results suggest that the hydrolysates of agricultural waste can be used as an assistant agent for PGPR to enhance their rhizosphere colonization and, hopefully, their plant beneficial function performance.

### 3.3 Effect of hydrolysate on SQR9-promoted maize growth

**Figure 6:**
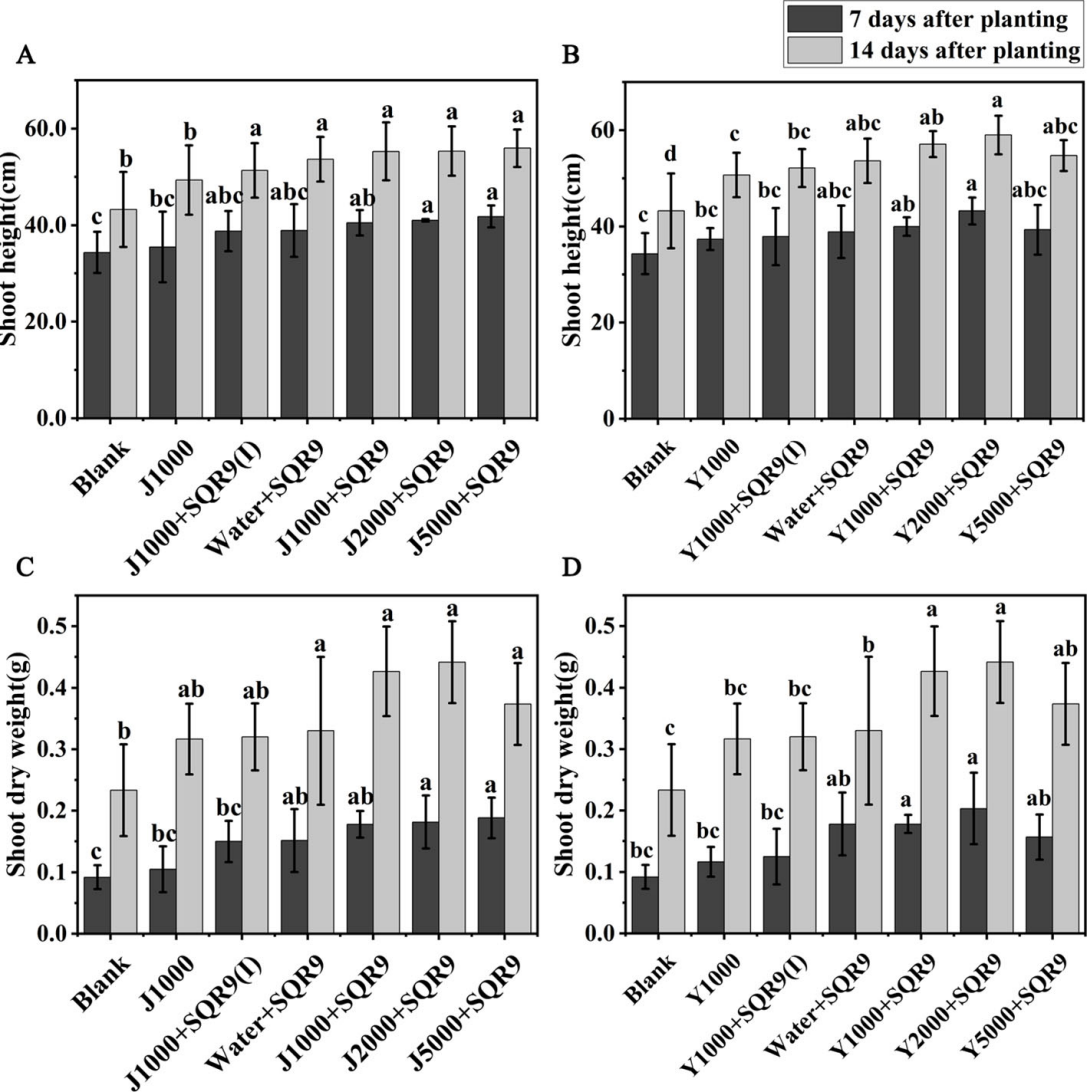
The promoting effects of different dilution treatments of hypoxic hydrolysates of mushroom bran **(A, C)** and tobacco waste **(B, D)** on maize growth. Significant differences among the different treatments are indicated by different letters (*p<* 0.05).

Strain SQR9 has been demonstrated as an efficient beneficial rhizobacterium for plant growth promotion (Shao et al., 2015). It is expected that enhancing SQR9’s rhizosphere colonization with the hypoxic hydrolysates of agricultural wastes will also enhance its ability to promote plant growth. Maize growth was investigated by measuring shoot height and shoot dry weight for the different treatments. The results showed that all treatments involving SQR9 promoted maize growth (**Figures 6A–D**). The application of diluted hypoxic hydrolysates of mushroom bran and tobacco waste materials, in addition to SQR9, further enhanced the growth-promoting effect on maize. Although there were no significant differences (ANOVA, *p* > 0.05) in shoot dry weight among the different dilution treatments of the hydrolysates, the 2000-fold dilution had the best effect (**Figures 6A–D**; **Supplementary Table S3**). These results suggest that the hydrolysates can be used with PGPR to improve its beneficial function.

## 4 Discussion

The yield of organic acids from hypoxic hydrolysate is influenced by various factors, including initial pH, temperature, inoculum size, moisture content, and fermentation time (Chen et al., 2013, 2007; Lee et al., 2014). The results indicate that the optimal initial pH and fermentation time for producing organic acids differ between mushroom bran and tobacco waste materials. To achieve higher levels of organic acid production, strategies such as enhancing hydrolysis to generate more soluble substrates and inhibiting methanogen activity are crucial. Methanogens consume organic acids, particularly acetic acid, to produce methane, creating an inverse relationship between their growth and the accumulation of organic acids (Yuan et al., 2006). When methanogen activity is inhibited, the concentration of organic acids can increase, leading to higher yields. Previous studies have indicated that the growth of methanogens is inhibited when the pH is less than 6.5, which favors the accumulation of organic acids (Fang and Liu, 2002). Wang et al. (2014) found that organic acid production at pH 6.0 was higher in anaerobic digestion of food waste, but Yuan et al. (2006) reported that short-chain fatty acid production from sludge was higher at pH values ranging from 8.0 to 11.0.

Temperature affects the production of organic acids mainly by influencing the growth of microorganisms, the activities of enzymes, and the hydrolysis rate (Shi et al., 2024). The optimum growth temperature for most anaerobic microorganisms is 35°C. The results of this study showed that the production of organic acids was highest at a temperature of 35°C for both mushroom bran and tobacco waste materials, which is consistent with the results of previous studies (Jiang et al., 2013). However, when considering the composition of VFAs, the optimal temperature varies. During anaerobic fermentation at 35°C and 45°C, acetic acid and propionic acid are the most produced acids, whereas butyric acid becomes the main product at 55°C (He et al., 2012). Nonetheless, our study does not extensively focus on the impact of temperature on the composition of VFAs, as all of these acids are key components in eliciting PGPR chemotaxis (Feng et al., 2019).

Hydrolytic bacteria and acidogenic bacteria are the two main functional microorganisms that promote VFA production. The inoculum is the fundamental driver that accelerates the process due to profuse anaerobic flora (Zhou et al., 2018). The number of functional bacteria depends on the inoculum concentration. Generally, a lower inoculum concentration contributes to acid accumulation within a suitable concentration range of 10% to 30%. We found that the production of VFAs was highest at an inoculum concentration of 15%, whether it was mushroom bran or tobacco waste materials (**Figures 3A, B**). This demonstrates that the inoculum concentration should not be too low, as it would result in an insufficient number of hydrolytic bacteria. However, if the concentration is too high, there may be a deficiency of nutrients available in the substrate limiting microbial activity.

Moisture content also affects substrate concentration. A high substrate concentration with low moisture content is advantageous for acid accumulation within the appropriate range, whereas too much moisture can inhibit microbial activity, resulting in a lower VFAs yield (Yellezuome et al., 2024). Our study showed that the VFAs yield was the highest when the moisture content was 85% (**Figures 3A, B**).

In theory, a longer fermentation time has advantages in producing VFAs from acidogenic fermentation because microorganisms have more time to react with the substrate (Zhang et al., 2024b). Peces et al. (2021) suggested that the highest VFAs yield occurred between 4 and 8 days. We observed the highest production of VFAs when the fermentation time was 168 h for mushroom bran and 144 h for tobacco waste materials (**Figures 3A, B**), which falls within the normal fermentation time.

The chemotactic response of PGPR to organic acids is dependent on the concentration of these acids. For instance, *Bacillus amyloliquefaciens* SQR9 exhibits a significant chemotactic reaction to 20 μmol l^-1^ of citric acid when the rhizosphere of cucumber is infected by pathogens (Liu et al., 2014). This indicates that the concentration of organic acids directly affects the chemotactic efficacy of PGPR. In our study, we observed that the optimal dilution for tobacco waste hydrolysate was 2000-fold, while for mushroom bran hydrolysate, it was 5000-fold (**Supplementary Figure S1**). Interestingly, the initial organic acid content of the tobacco waste hydrolysate was higher than that of the mushroom bran hydrolysate (**Figures 3A, B**).

This disparity may reflect the different types and bioactive effects of organic acids present in the two hydrolysates. Specifically, the mushroom bran hydrolysate might contain more effective organic acids, such as citric acid, that have a more pronounced chemotactic effect on SQR9. Citric acid is not only a common plant root exudate but it also plays a crucial role in attracting and colonizing PGPR (Zhang et al., 2023). In contrast, although the tobacco waste hydrolysate has a higher overall organic acid content, these acids may not be as effective as those in the mushroom bran hydrolysate, or their combination may not be as conducive to eliciting a chemotactic response from SQR9.

Furthermore, the different chemotactic responses may be related to the molecular structure and functional properties of the organic acids (Zhang et al., 2024a). Small molecule organic acids, such as citric acid might be more easily detected by SQR9, triggering a stronger chemotactic response. In contrast, larger or more complex organic acids might require higher concentrations or specific environmental conditions to produce a similar effect (Feng et al., 2022). This could explain why SQR9 shows a significant chemotactic response in mushroom bran hydrolysate even at higher dilution ratios (5000-fold).

The research demonstrates significant commercial potential in the hypoxic hydrolysis of mushroom bran and tobacco waste materials to produce organic acids, which can be utilized to enhance plant growth and support beneficial microbial colonization. This approach offers several key advantages, including the development of eco-friendly biofertilizers that improve plant health by fostering PGPR colonization. It also provides a sustainable solution for waste management by converting agricultural and industrial byproducts into valuable products.

However, commercializing this technology involves addressing several challenges. Scaling up from laboratory to industrial production requires effective process control and consistency, which can be managed by implementing advanced fermentation technologies and conducting pilot studies. Cost efficiency is another concern, as the initial production and setup costs may be high. Solutions include optimizing the fermentation process to lower costs and exploring less expensive raw material options. Ensuring product stability and quality is crucial, which necessitates strict quality control measures and standardized procedures. Market acceptance may also be a barrier, as new products often face skepticism. Overcoming this involves educating the market, providing samples, and showcasing the benefits through case studies.

Compared to traditional chemical fertilizers, this method has advantage due to its environmental benefits and the use of waste materials, aligning with sustainability goals. Although it may involve a more complex production process and higher initial costs, it offers long-term benefits by improving soil health and plant resilience. Overall, the research presents a promising alternative to conventional fertilizers, with the potential to boost agricultural productivity and support sustainable waste management practices. By addressing the outlined challenges and leveraging the unique benefits, this technology could be effectively integrated into the market, offering both environmental and economic advantages.

This study has several limitations. Firstly, the specific organic acid components in the hypoxic hydrolysate were not effectively analyzed, resulting in an unclear understanding of the key chemotactic substances for PGPR. This uncertainty limits the ability to further optimize the fermentation process. Secondly, although the chemotactic response of SQR9 to the hypoxic hydrolysate was observed in laboratory experiments, the molecular mechanisms by which the hydrolysate induces SQR9 colonization in the maize rhizosphere remain unexplored. Additionally, since this study is based on pot experiments, the future application of PGPR in field trials is crucial. In actual agricultural settings, the effectiveness of PGPR may be affected by various soil types and environmental conditions. Therefore, additional field studies will be essential to validate the findings from laboratory experiments and evaluate the practical potential of this technology in real-world agricultural production.

## 5 Conclusion

Hypoxic hydrolysis of mushroom bran and tobacco waste was used to produce organic acids. Optimal conditions varied slightly between the two: mushroom bran needed pH 8.0, 40°C, 15% inoculum, 85% moisture, and 168 hours, while tobacco waste required pH 6.0, 40°C, 15% inoculum, 85% moisture, and 144 hours. Diluting the hydrolysates (5000-fold for mushroom bran and 2000-fold for tobacco waste) boosted the growth-promoting effects of the PGPR strain SQR9 in maize. This suggests that hypoxic fermentation can turn agricultural waste into effective microbial fertilizers, reducing reliance on chemical alternatives and promoting sustainable farming.

## Data Availability

The original contributions presented in the study are included in the article/**Supplementary Material**. Further inquiries can be directed to the corresponding author.
