# Supplementary material for: Hydrolysis products of agricultural waste can serve as microbial fertilizer enhancers to promote the growth of maize crops
Source: Front Plant Sci. 2024 Oct 17;15:1405527. doi: 10.3389/fpls.2024.1405527 (PMC11524819; doi:10.3389/fpls.2024.1405527)
Supplement: Supplementary file 1 [file DataSheet1.docx]

**Hydrolysis products of agricultural waste can serve as microbial fertilizer enhancers to promote the growth of maize crops**

Yu Xu^1^**^†^**, Wei Wang^2^**^†^**, He Wang^1^, Yinping Tian^1^, Zhengfu Yue^3*^, Cheng Li^4^, Yuefeng Wang^4^, Jing Zhang^5^, Ruifu Zhang^2^

^1^Key Laboratory of Agricultural Water Resources, Hebei Key Laboratory of Soil Ecology, Center for Agricultural Resources Research, Institute of Genetics and Developmental Biology, Chinese Academy of Sciences, Shijiazhuang 050021, China

^2^Jiangsu Provincial Key Lab for Organic Solid Waste Utilization, National Engineering Research Center for Organic-based Fertilizers, Jiangsu Collaborative Innovation Center for Solid Organic Waste Resource Utilization, Nanjing Agricultural University, Nanjing, 210095, P.R. China

^3^Key Laboratory of Low-carbon Green Agriculture in Tropical region of China, Ministry of Agriculture and Rural Affairs; Hainan Key Laboratory of Tropical Eco-Circular Agriculture, Environment and Plant Protection Institute, Chinese Academy of Tropical Agricultural Sciences, Haikou 571101, China

^4^Hebei Provincial Laboratory of Water Environmental Science, Hebei Provincial Academy of Ecological and Environmental Sciences, Shijiazhuang 050037, China.

^5^Department of Environmental Sciences, School of Tropical and Laboratory Medicine, Hainan Medical University, Haikou 571199, China

^*^**Corresponding author:** Zhengfu Yue

E-mail: yuezhengfu2011@163.com, Tel: +86-898-6696-9275, Fax: +86-898-6696-9211

**^†^**These authors contributed equally to this work.

**Methods S1 Chemotaxis Assay**

SQR9 was cultured in LB broth at 35 °C and 170 rpm for approximately 8 hours to prepare the seed culture. This seed culture was then inoculated at 1% (v/v) into 100 mL of fresh LB broth in a flask and further incubated at 35 °C and 170 rpm until the OD_600_ reached 0.5-0.6. The cells were harvested by centrifugation at 6000 rpm for 10 minutes at room temperature and washed twice with chemotaxis buffer (100 mM potassium phosphate buffer, 20 μM EDTA, pH 7.0). The cell pellet was then resuspended in an equal volume of chemotaxis buffer.

The qualitative chemotaxis assay was performed using the drop assay method described by Grimm and Harwood (Grimm and Harwood, 1997). A mixture of 12 mL of the bacterial suspension and 3 mL of 1% hydroxypropyl methylcellulose was prepared to reduce the diffusion of the attractant. The mixture was gently shaken to ensure homogeneity. Then, 3 mL of the mixture was transferred to a 60 mm cell culture dish, ensuring no air bubbles were present. A 10 μL drop of each treatment solution was carefully placed in the center of the bacterial suspension. Sterile water was used as a blank control. The formation of a chemotactic ring around the drop was observed to determine chemotactic activity.

**Results S1 Chemotactic response of** **SQR9 to diluted anaerobic fermentation liquids**

The 1000-fold and 2000-fold dilutions of hypoxia hydrolysate of mushroom bran did not exhibit any chemotactic rings, whereas the 5000-fold dilution showed a distinct chemotactic ring. This indicated that SQR9 exhibited significant chemotactic activity towards the 5000-fold dilution of hypoxia hydrolysate of mushroom bran. In contrast, chemotactic rings were observed in both the 1000-fold and 2000-fold dilutions of hypoxia hydrolysate of tobacco waste, with the 2000-fold dilution showing a more pronounced ring. No chemotactic ring was observed in the 5000-fold dilution. These results suggested that SQR9 had significant chemotactic activity towards the 2000-fold dilution of hypoxia hydrolysate of tobacco waste.


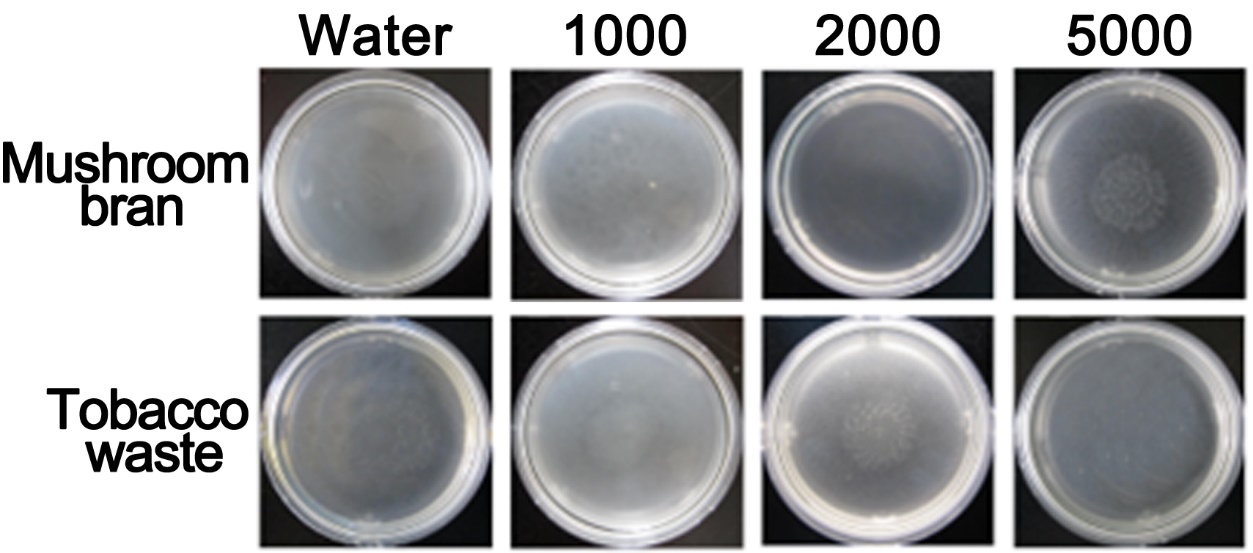


**Fig. S1** Chemotactic response of SQR9 to diluted anaerobic fermentation liquids

**Table S1** Orthogonal experimental design for acid production via hypoxic hydrolysis of mushroom bran

| No. | Combination | Experimental factors | | | | |
| --- | --- | --- | --- | --- | --- | --- |
|  |  | pH | Temperature/℃ | Inoculum concentration/% | Moisture content/% | Fermentation time/h |
| 1 | A1B1C1D1E1 | 7 | 35 | 10 | 85 | 156 |
| 2 | A1B1C2D2E2 | 7 | 35 | 15 | 90 | 168 |
| 3 | A1B2C1D1E2 | 7 | 40 | 10 | 85 | 168 |
| 4 | A1B2C2D2E1 | 7 | 40 | 15 | 90 | 156 |
| 5 | A2B1C1D2E2 | 8 | 35 | 10 | 90 | 168 |
| 6 | A2B1C2D1E1 | 8 | 35 | 15 | 85 | 156 |
| 7 | A2B2C1D2E1 | 8 | 40 | 10 | 90 | 156 |
| 8 | A2B2C2D1E2 | 8 | 40 | 15 | 85 | 168 |

**Table S2** Orthogonal experimental design for acid production via hypoxic hydrolysis of tobacco waste

| No. | Combination | Experimental factors | | | | |
| --- | --- | --- | --- | --- | --- | --- |
|  |  | pH | Temperature/℃ | Inoculum concentration/% | Moisture content/% | Fermentation time/h |
| 1 | A1B1C1D1E1 | 5 | 35 | 10 | 85 | 132 |
| 2 | A1B1C2D2E2 | 5 | 35 | 15 | 90 | 144 |
| 3 | A1B2C1D1E2 | 5 | 40 | 10 | 85 | 144 |
| 4 | A1B2C2D2E1 | 5 | 40 | 15 | 90 | 132 |
| 5 | A2B1C1D2E2 | 6 | 35 | 10 | 90 | 144 |
| 6 | A2B1C2D1E1 | 6 | 35 | 15 | 85 | 132 |
| 7 | A2B2C1D2E1 | 6 | 40 | 10 | 90 | 132 |
| 8 | A2B2C2D1E2 | 6 | 40 | 15 | 85 | 144 |

**Table S3** ANOVA table in different treatment

| Material | Condition | F value | P value | Significance |
| --- | --- | --- | --- | --- |
| Mushroom bran | pH | 88.5 | 1.78E-06 | *** |
|  | Temperature | 154 | 2.02E-07 | *** |
|  | Inoculum | 3942 | 5.14E-13 | *** |
|  | Moisture | 4208 | 3.96E-13 | *** |
|  | Time | 1864 | 4.15E-09 | *** |
|  | Orthogonal | 916 | 2.20E-16 | *** |
|  | SQR9 number (7d) | 44.4 | 2.55E-08 | *** |
|  | SQR9 number (14d) | 9.29 | 3.21E-04 | *** |
|  | Stem dry weight (7d) | 3.64 | 2.17E-02 | * |
|  | Stem dry weight (14d) | 2.01 | 1.32E-01 |  |
| Tobacco waste | pH | 489 | 2.12E-09 | *** |
|  | Temperature | 2679 | 2.40E-12 | *** |
|  | Inoculum | 2955 | 1.62E-12 | *** |
|  | Moisture | 6363 | 7.58E-14 | *** |
|  | Time | 6474 | 9.93E-11 | *** |
|  | Orthogonal | 951 | 2.20E-16 | *** |
|  | SQR9 number (7d) | 15.7 | 1.72E-05 | *** |
|  | SQR9 number (14d) | 14.3 | 2.95E-05 | *** |
|  | Stem dry weight (7d) | 2.43 | 8.05E-02 |  |
|  | Stem dry weight (14d) | 2.06 | 1.25E-01 |  |
